# Influence of gender and sexual hormones on outcomes after pituitary surgery: a systematic review and meta-analysis

**DOI:** 10.1007/s00701-023-05726-z

**Published:** 2023-08-09

**Authors:** Sven Theiler, Saskia Hegetschweiler, Victor E. Staartjes, Antonio Spinello, Giovanna Brandi, Luca Regli, Carlo Serra

**Affiliations:** 1 Machine Intelligence in Clinical Neuroscience (MICN) Laboratory, Department of Neurosurgery, Clinical Neuroscience Center, University Hospital Zurich, University of Zurich, Frauenklinikstrasse 10, 8091 Zurich, Switzerland; 2 Institute for Intensive Care, University Hospital Zurich, University of Zurich, Zurich, Switzerland

**Keywords:** Pituitary, Adenoma, Surgery, Gender, Male, Female

## Abstract

**Background:**

Although there is an increasing body of evidence showing gender differences in various medical domains as well as presentation and biology of pituitary adenoma (PA), gender differences regarding outcome of patients who underwent transsphenoidal resection of PA are poorly understood. The aim of this study was to identify gender differences in PA surgery.

**Methods:**

The PubMed/MEDLINE database was searched up to April 2023 to identify eligible articles. Quality appraisal and extraction were performed in duplicate.

**Results:**

A total of 40 studies including 4989 patients were included in this systematic review and meta-analysis. Our analysis showed odds ratio of postoperative biochemical remission in males vs. females of 0.83 (95% CI 0.59–1.15, *P* = 0.26), odds ratio of gross total resection in male vs. female patients of 0.68 (95% CI 0.34–1.39, *P* = 0.30), odds ratio of postoperative diabetes insipidus in male vs. female patients of 0.40 (95% CI 0.26–0.64, *P* < 0.0001), and a mean difference of preoperative level of prolactin in male vs. female patients of 11.62 (95% CI − 119.04–142.27, *P* = 0.86).

**Conclusions:**

There was a significantly higher rate of postoperative DI in female patients after endoscopic or microscopic transsphenoidal PA surgery, and although there was some data in isolated studies suggesting influence of gender on postoperative biochemical remission, rate of GTR, and preoperative prolactin levels, these findings could not be confirmed in this meta-analysis and demonstrated no statistically significant effect. Further research is needed and future studies concerning PA surgery should report their data by gender or sexual hormones and ideally further assess their impact on PA surgery.

**Supplementary Information:**

The online version contains supplementary material available at 10.1007/s00701-023-05726-z.

## Introduction

Pituitary adenoma (PA) is the second most frequent intracranial neoplasm and presents clinically as an incidental finding or with endocrine or mass effect manifestations [4]. Over the past decades, transsphenoidal surgery has established itself as the gold standard, first-line treatment for most subtypes of PA [15, 45, 82].

Recently, the influence of gender on clinical outcomes has seen a massive increase in interest among the scientific community, as outcome differences with clinical relevance have been established in various domains such as cardiovascular disease, autoimmune disease, and infectious disease. [11, 20, 41, 49, 50, 56, 62, 63, 65, 70, 80, 85, 87, 90]

Although there is literature concerning gender differences in the biology of the pituitary gland and in the presentation as well as the biology of PA, gender differences regarding the outcome of patients who underwent transsphenoidal resection of PA are poorly understood. [3, 25, 61, 63, 74, 76, 77, 88] It is currently unknown whether the different physiological hormone levels or any other gender differences may impact patient selection, success of treatment, or hormonal cut-off values [28, 32, 33].

Published data on this topic are scarce, and the authors are not aware of any literature review on gender differences in pituitary surgery, although there is one study assessing gender differences in non-surgical aspects of non-functioning PA (NFPA) [25]. Systematic reviews and meta-analyses can lead to more realistic results with better generalisability and less risk of bias compared to single studies [64]. In this study, we systematically reviewed the literature to evaluate the influence of gender and sexual hormones on outcomes after endoscopic or microscopic transsphenoidal PA surgery.

## Materials and methods

### Overview

A systematic review was carried out to identify any studies reporting at least one of (1) GTR (rate of radiological gross total resection), (2) rate of new endocrinological deficits, or (3) biochemical remission (for patients with hormone-secreting adenomas) after resection of PA stratified by gender or by preoperative sexual hormone (estrogen, testosterone, prolactin). Title and abstract screening, full-text review, and data extraction were handled independently by two reviewers (ST and SH), and disagreements at any stage were resolved by discussion and consensus. Persisting disagreements were resolved by discussion with a third reviewer (VS). We followed the Preferred Reporting Items for Systematic Reviews and Meta-Analyses (PRISMA) protocol [64].

### Search strategy

The PubMed/MEDLINE database was searched to identify eligible articles. The search strategy included combinations of the following terms: pituitary; adenoma; surgery; resection; transsphenoidal; gender; sex; male; female; prolactin; testosterone; estrogen; gross total resection; GTR; deficit; endocrine; endocrinological; and biochemical (Supplementary Table 2). Word variations and exploded medical subject headings were searched for whenever feasible. Additionally, reference lists were hand-searched to identify further studies of interest. The last comprehensive search was conducted on April 30, 2023.

### Study selection

Only in vivo studies enrolling humans of all age groups in English, Italian, French, Dutch, and German were considered. As no controlled trials were anticipated, prospective and retrospective single-arm cohort studies and case series of adult individuals were also included. We excluded pediatric cases series. Case reports and small case series with less than 5 patients were excluded. To be considered, patients had to undergo endoscopic or microscopic transsphenoidal resection of PA. Studies had to assess at least one of the three abovementioned outcomes of interest stratified either by gender or by sexual hormone levels. In this way, we were able to rate the potential influence of sexual hormones and gender on outcomes. Studies reporting only resection of Rathke cleft cysts, craniopharyngiomas, or other lesions were excluded. We also excluded studies dealing mainly with transcranial or combined procedures. Studies reporting the outcomes of interest with a mix of targeted GTR and subtotal resection (STR) (i.e., a realistic caseload) were included. Exact cohort duplicates were excluded, although we did include updates of previously published cohorts with a sample size increase of at least 50%. Studies published before the 1st of January 1990 were excluded.

### Data extraction and quality assessment

We extracted the following information if available from all included publications: study design and year of publication, number of patients, mean patient age and gender distribution, data on prolactin, testosterone, and estrogen levels, as well as data on GTR, new endocrinological deficits, and biochemical remission among patients with secreting adenomas. The methodological quality of included studies was graded using the GRADE framework [38].

### Statistical meta-analysis

Based on anticipated heterogeneity and low event rates among studies, a random-effects analysis model (Mantel–Haenszel) that assesses odds ratios (OR) was chosen as the primary statistical method [39]. Cochran’s *Q* and *I*^2^ were used to evaluate heterogeneity, and a *P* < 0.1 was considered as relevant heterogeneity. All statistical analyses were carried out in RevMan version 5.4. Forest plots were generated to illustrate the main results of the meta-analysis.

## Results

### Literature search

**Fig. 1:**
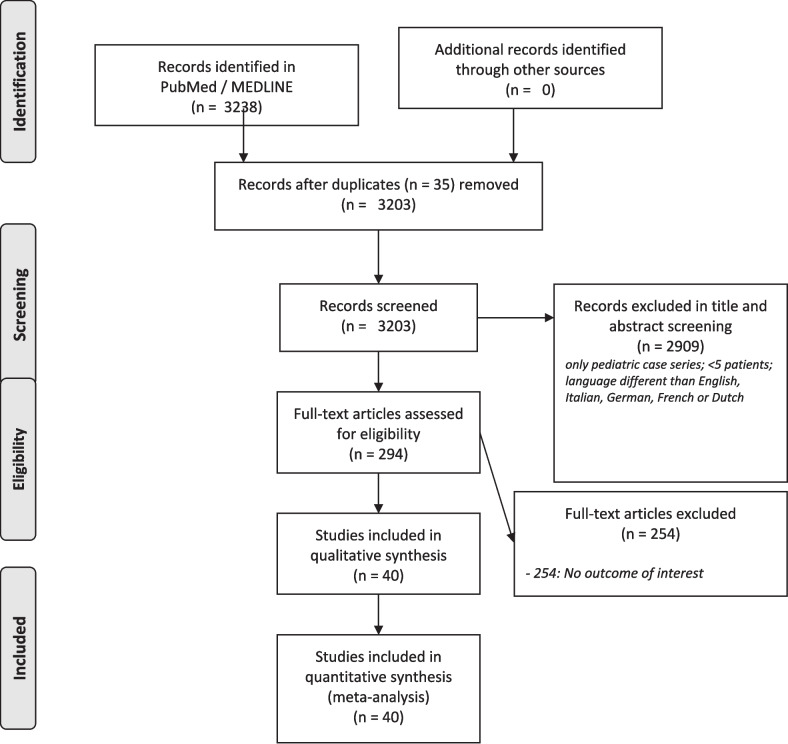
Flowchart of the literature eligibility assessment process

As seen in the PRISMA flowchart in Fig. 1, the PubMed/Medline search provided 3238 articles to which none were added through other sources. After duplicate removal (*n* = 35), 3203 records were screened and 294 were assessed for eligibility through full-text screening, concluding 40 studies included in qualitative synthesis, all of which were also eligible for quantitative meta-analysis [2, 6–10, 12, 14, 17, 21, 27, 29, 31, 34, 40, 42, 47, 48, 51–53, 55, 57–59, 67, 68, 75, 78, 79, 81, 83, 86, 89, 91–96].

### Study characteristics

The details of the 40 included studies are summarized in Table 1. We identified 31 studies reporting postoperative biochemical remission [2, 6, 8, 10, 12, 14, 21, 29, 31, 34, 40, 42, 47, 48, 51–53, 55, 57–59, 67, 68, 75, 78, 79, 81, 86, 89, 92, 94], eight reporting rate of GTR [7, 17, 27, 68, 75, 91, 94, 95], five reporting incidence of postoperative diabetes insipidus (DI) [7, 21, 58, 83, 93], four reporting preoperative levels of prolactin [21, 67, 94, 96], one study reporting rate of postoperative hyperprolactinemia [94], one study reporting both postoperative adrenocorticotropic hormone (ACTH) and thyroid-stimulating hormone (TSH) deficiency [9], one study reporting postoperative panhypopituitarism [83], and one study reporting any endocrinological deficit [55], while each of those endpoints was stratified by gender. No studies were found that reported preoperative levels of testosterone or estrogen, postoperative follicle-stimulating hormone (FSH), luteinizing hormone (LH) deficiency, growth hormone (GH) deficiency, or postoperative rate of syndrome of inappropriate antidiuretic hormone secretion (SIADH) by gender. Endpoints reported by one or less study could not be analyzed and are reported in Supplementary Table 1.

**Table 1:** Details of selected studies

Year	Study design	First author	Journal	No. total	No. male	No. female	Age mean/median	Male preprolactin (ng/ml)	Female preprolactin (ng/ml)	Male postGTR	Female postGTR	Male diab. insip	Female diab. insip	No. secreting tumors	Male biochem remission	Female biochem remission	GRADE
2018	Retrospective	Antunes	International Journal of Basic and Clinical Endocrinology	69	36	33	Mean: 45							69	13	18	Very low (limitation: sample size)
2020	Retrospective	Asha	Journal of Neurosurgery (JNS)	81	36	45	Mean: 45.1							81	28	31	Very low (limitation: sample size)
2019	Retrospective	Aydin	World Neurosurgery	27	16	11	Mean 44.7			9	6	1 permanent 1 transient	2 transient				Very low (limitation: sample size)
2020	Retrospective	Aydin	Clinical Neurology & Neurosurgery	95	39	56	Mean: 43.4							95	24	39	Very low (limitation: sample size)
2010	Retrospective	Baumann	Neurosurgical Review	6	5	1	Mean 46										Very low (limitation: sample size)
2010	Retrospective	Bellut	Journal of Neurosurgery (JNS)	37	24	13	Mean: 47							37	16	10	Very low (limitation: sample size)
2020	Retrospective	Bora	World Neurosurgery	85	26	59	Mean: 28							85	14	45	Very low (limitation: sample size)
2010	Retrospective	Campbell	Journal of Neurosurgery (JNS)	26	14	12	Mean: 45.7							26	11	4	Very low (limitation: sample size)
2022	Retrospective	Chen	Frontiers in Endocrinology	239	102	137	Mean: 51.12			18	28						Low
2016	Retrospective	Cote	Neurosurgery	7	5	2	Mean 51.6	11.46 (SD 5.99)	84.4 (SD 54.45)			1 permanent	1 transient	7	4	2	Very low (limitation: sample size)
2019	Retrospective	Fallah	World Neurosurgery	80	61	19	Mean: 46.8			49	17						Very low (limitation: sample size)
2009	Retrospective	Fomekong	Clinical Neurology & Neurosurgery	40	3	37	Mean 43							40	1	31	Very low (limitation: sample size)
2021	Retrospective	Giese	Experimental and Clinical Endocrinology & Diabetes	162	48	114	Mean: 32.4							162	23	87	Low
2022	Prospective	Guo	Journal of Neurosurgery (JNS)	659	293	366	Mean: 41.7							659	127	118	Low
2010	Prospective	Hofstetter	Journal of Neurosurgery (JNS)	24	13	11	Mean: 50.7							24	6	5	Very low (limitation: sample size)
2011	Retrospective	Jane	Journal of Clinical Endocrinology & Metabolism (JCEM)	60	33	27	Mean: 48							60	23	19	Very low (limitation: sample size)
2017	Retrospective	Kim	World Neurosurgery	134	60	74	Mean: 46							134	55	44	Low
2020	Retrospective	Kim	Journal of Neurosurgery (JNS)	31	13	18	Mean: 41.5							31	10	16	Very low (limitation: sample size)
2012	Retrospective	Ku	Operative Neurosurgery	282	121	161	Mean: 41.8							282	97	113	Low
2016	Prospective	Ku	Medicine	187	83	104	Median: 42							187	75	82	Low
2022	Retrospective	Lasolle	Annales d'Endocrinologie	60	11	49	Median: 26							60	5	41	Very low (limitation: sample size)
2019	Prospective	Little	Journal of Neurosurgery (JNS)	169	99	70	Mean: 57.6							95 (m: 66, f: 29)	13	7	Very low (limitation: sample size)
2020	Retrospective	Lyu	Medicine	214	102	112	Mean: 48.86							93 (m: 29, f: 64)	24	54	Low
1997	Retrospective	Mason	Journal of Neurosurgery (JNS)	10	5	5	Mean: 32.5					1 permanent	3 transient	10	3	4	Very low (limitation: sample size)
2017	Retrospective	Mayberg	Journal of Neurosurgery (JNS)	81	14	67	Mean: 38.1							81	9	48	very low (limitation: sample size)
2022	Retrospective	Osorio	Journal of Neurosurgery (JNS)	219	78	141	Mean: 35	1306.4 (SD 2444.1)	445.8 (SD 1385)					219	38	108	Low
2017	Retrospective	Park	Journal of Clinical Endocrinology & Metabolism (JCEM)	463	203	260	Mean: 42.9			188	223			463	128	175	Low
2000	Retrospective	Sanno	Journal of Neurosurgery (JNS)	16	4	12	Mean: 41.2			1	7			16	2	8	Very low (limitation: sample size)
2019	Retrospective	Serban	Neuroendocrinology	61	12	49	Mean: 41							61	10	41	Very low (limitation: sample size)
2017	Retrospective	Shin	World Neurosurgery	50	11	39	Median: 40							50	6	29	Very low (limitation: sample size)
2018	Prospective	Taghvaei	World Neurosurgery	68	36	32	Mean: 39.35							68	25	19	Very low (limitation: sample size)
2022	Retrospective	Tiwari	Cureus	210	104	106	Mean: 52.3					5	11				Low
2019	Retrospective	Wang	World Neurosurgery	87	34	53	Mean: 39.7							87	25	26	Very low (limitation: sample size)
2013	Retrospective	Wilson	Pituitary	14	6	8	Mean: 41.4							14	3	5	Very low (limitation: sample size)
2022	Retrospective	Xu	Current Medical Science	144	72	72	Mean: 50			35	56						Low
2015	Retrospective	Yamada	International Journal of Basic and Clinical Endocrinology	252	37	215	Median: 42.7							230 (m: 32, f: 198)	25	161	Very low (limitation: sample size)
2023	Retrospective	Yasuda	World Neurosurgery	344	110	234	Mean: 46.47					23 transient	93 transient				Low
2018	Retrospective	Yoo	Journal of Neurological Surgery	79	22	57	Mean: 36.8	2281.0 (SD 4715.82)	532.9 (SD 1213.87)	15	53			79	13	52	Very low (limitation: sample size)
2021	Retrospective	Zhu	Clinical Neurology & Neurosurgery	107	38	69	Mean: 46.7			12	14						Very low (limitation: sample size)
2015	Retrospective	Zieliński	Endokrynologica Polska	10	3	7	Mean: 48	9.57 (SD 10.02)	14.4 (SD 14.36)								Very low (limitation: sample size)

*No. total* total number of patients, *No. male* total number of male patients, *No. female* total number of female patients, *Male preProlactin (ng/ml)* preoperative levels of prolactin in male patients in ng/ml, *Female preProlactin (ng/ml)* preoperative levels of prolactin in female patients in ng/ml, *Male postGTR* number of male patients with gross total resection, *Female postGTR* number of female patients with gross total resection, *Male diab. insip.* number of male patients with postoperative permanent or transient diabetes insipidus, *Female diab. insip*. number of female patients with postoperative permanent or transient diabetes insipidus, *Male biochem remission* number of male patients with postoperative biochemical remission, *Female biochem remission* number of female patients with postoperative biochemical remission, *GRADE* methodological quality of included studies graded by the GRADE framework

Detailed qualitative interpretation of all analyzed outcomes including detailed certainty assessments is shown in Table 2.

**Table 2:** Summary of findings

Certainty assessment							No. of patients		Effect		Certainty	Importance
No. of studies	Study design	Risk of bias	Inconsistency	Indirectness	Imprecision	Other considerations	Male	Female	Relative (95% CI)	Absolute (95% CI)		
Biochemical remission												
31	Observational studies	Not serious	Serious^a^	Not serious	Serious^a^	All plausible residual confounding would reduce the demonstrated effect	856/1410 (60.7%)	1442/2195 (65.7%)	**OR 0.83** (0.59 to 1.15)	**43 fewer per 1.000** (from 126 fewer to 31 more)	⨁◯◯◯ very low	Not important
GTR												
8	Observational studies	Not serious	Serious^a^	Not serious	Very serious^a^	All plausible residual confounding would reduce the demonstrated effect	327/518 (63.1%)	404/637 (63.4%)	**OR 0.68** (0.34 to 1.39)	**93 fewer per 1.000** (from 263 fewer to 73 more)	⨁◯◯◯ Very low	Not important
Diabetes insipidus												
5	Observational studies	Not serious	Not serious	Not serious	Serious^b^	All plausible residual confounding would reduce the demonstrated effect	32/240 (13.3%)	110/358 (30.7%)	**OR 0.40** (0.26 to 0.64)	**157 fewer per 1.000** (from 204 to 86 fewer)	⨁⨁◯◯ Low	Not important
Prolactin												
4	Observational studies	Not serious	Very serious^c^	Not serious	Extremely serious^c^	All plausible residual confounding would reduce the demonstrated effect	108	207	-	MD **11.62 higher** (119.04 lower to 142.27 higher)	⨁◯◯◯ Very low	Not important

*CI* confidence interval, *MD* mean difference, *OR* odds ratio

Explanations:

^a^High heterogeneity of included studies, most included studies had a relatively low sample size

^b^Low sample size of most included studies, relatively low number of patients overall

^c^High heterogeneity, low sample size, sensitivity of meta-analyses of mean differences towards non-normal distributions of the source data, which could not be judged from the original publications

### Biochemical remission

**Fig. 2:**
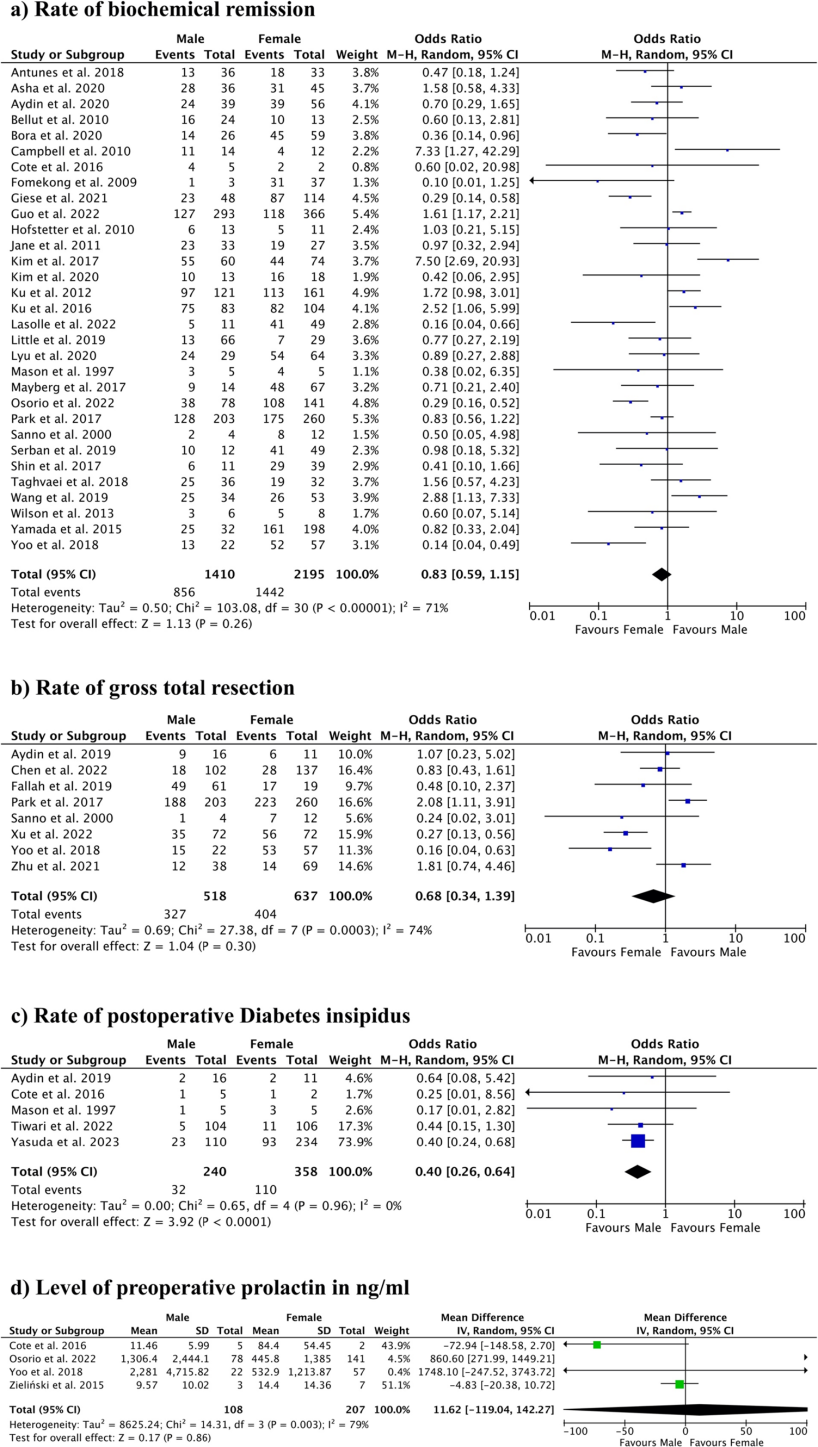
Results of random-effects meta-analysis. **a** Rate of biochemical remission. **b** Rate of gross total resection. **c** Rate of postoperative diabetes insipidus. **d** Level of preoperative prolactin in ng/ml

Overall, 31 studies including 3605 patients (1410 male, 2195 female) were analyzed via random-effects meta-analysis, which showed an odds ratio of postoperative biochemical remission in males vs. females of 0.83 (95% CI 0.59–1.15). Heterogeneity was high with a *I*^2^-value of 71% (*P* < 0.00001) and the overall effect was 1.13 (*P* = 0.26) (Fig. 2). Twenty-three of the included studies were rated at a GRADE certainty of “very low” due to their small sample size while six were rated at a GRADE certainty of “low” (Table 1). Overall, after a detailed certainty assessment, the certainty of the outcome of this analysis was very low (Table 2).

### Rate of GTR

In the evaluation of eight studies including 1155 patients (518 male, 637 female) via random-effects meta-analysis, an odds ratio of GTR in male vs. female patients showed to be 0.68 (95% CI 0.34–1.39). Again, heterogeneity was high with a *I*^2^-value of 74% (*P* = 0.0003) and the overall effect was 1.04 (*P* = 0.30) (Fig. 2). Five of the included studies were rated at a GRADE certainty of “very low” due to their small sample size while three were rated at a GRADE certainty of “low” (Table 1). Overall, after a detailed certainty assessment, the certainty of the outcome of this analysis was very low (Table 2).

### Postoperative diabetes insipidus

The rate of postoperative DI in male vs. female patients was analyzed via random-effects meta-analysis including five studies with 598 patients (240 male, 358 female). The odds ratio was shown to be 0.40 (95% CI 0.26–0.64) with a low heterogeneity with a *I*^2^-value of 0% (*P* = 0.96). The overall effect was 3.92 (*P* < 0.0001) (Fig. 2). Three of the included studies were rated at a GRADE certainty of “very low” due to their small sample size while two were rated at a GRADE certainty of “low” (Table 1). Overall, after a detailed certainty assessment, the certainty of the outcome of this analysis was low (Table 2).

### Preoperative level of prolactin

Four studies including 315 patients (108 male, 207 female) were analyzed via random-effects meta-analysis. The mean difference in the preoperative level of prolactin in male vs female patients was shown to be 11.62 (95% CI − 119.04–142.27) with high heterogeneity of *I*^2^ = 79% (*P* = 0.003). The overall effect was shown to be 0.17 (*P* = 0.86) (Fig. 2). Three of the included studies were rated at a GRADE certainty of “very low” due to their small sample size while one was rated at a GRADE certainty of “low” (Table 1). Overall, after a detailed certainty assessment, the certainty of the outcome of this analysis was very low (Table 2).

## Discussion

The aim of this study was to identify gender differences in PA surgery. In our meta-analysis, there was a significantly higher rate of postoperative DI in female patients after endoscopic or microscopic transsphenoidal PA surgery, and although there was some data in isolated studies suggesting the influence of gender on postoperative biochemical remission, rate of GTR, and preoperative prolactin levels in patients after endoscopic or microscopic transsphenoidal PA surgery, these findings could not be confirmed in this meta-analysis and demonstrated no statistically significant effect of gender after endoscopic or microscopic PA surgery.

Gender differences are an important and established influence on clinical outcomes in various domains. [41, 50, 62, 63, 65, 70] While there is evidence of gender differences in clinical presentation and tumor size of PA [3, 16, 19, 25, 44, 68, 73, 84, 94], there is still little data available on its influence in PA surgery. Furthermore, in published studies, few data on baseline characteristics and outcomes concerning gender and sexual hormone status of participants is included: preoperative levels of testosterone or estrogen, postoperative FSH, LH or GH deficiency, or postoperative frequency of SIADH are not reported in any study and only single studies reported postoperative hyperprolactinemia [94], postoperative panhypopituitarism [83], postoperative ACTH and TSH deficiency [9], and any endocrinological deficit [55] stratified by gender (Supplementary Table 1).

In our analysis, we found overall no statistically significant impact of gender on biochemical remission of PA after surgery (Fig. 1). In single studies, however, differences by sex on biochemical remission are reported: Park et al. (2017) [68] found worse outcomes of male patients in comparison to premenopausal female patients with GH secreting adenomas. In addition, Yoo et al. [94] found worse outcomes in male than female patients with prolactinomas, and Arasho et al. [3] reported a worse outcome in male than female patients with prolactinoma, although in patients with non-functioning PA, significantly worse outcomes were observed in female than in male patients. The reasons for both these findings remain unclear but might be explained due to the difference in the distribution of patients with female patients with prolactinoma typically presenting at a lower age while not only older age at presentation but also larger tumor size and a possibly more aggressive biology of male patients with prolactinoma have been discussed, which in turn might lead to better outcomes in female patients [1, 13, 18, 19, 23, 24, 26, 94]. Although in prolactinoma a difference in the distribution of patients may be the reason for gender-specific outcomes, in other subtypes of PA the reason for such a difference, as in single studies there seems to be, remains unclear and future studies are needed to not only fully establish such a difference, but also a possible reason for it [3, 68]. The findings of these single studies suggest that in specific subtypes of PA and in specific age groups, sex and gender might have an impact on the biochemical remission of PA after surgery.

The impact of gender on GTR also showed no statistically significant difference between male and female patients. These findings are consistent with the findings of Park et al. (2017) [68], where no statistically significant influence of gender on the rate of GTR was found. Contrary to these findings, Yoo et al. (2018) [94] reported a significantly lower number of GTR in male patients, although this study included a much lower sample size than the analysis of this study and the study of Park et al. [68].

In our analysis, we found a significantly higher rate of postoperative DI in female than male patients. With a low heterogeneity of our analysis, these findings are consistent with included studies. It should be mentioned that there is one study by Joshi et al. [43] which specified postoperative DI by gender for all transsphenoidal surgery and, when analyzing for PA surgery alone, did not find a statistically significant difference of gender on postoperative DI and did not discuss this in detail. Although the rate of postoperative DI in female patients was significantly higher in our analysis, the possible reason for this outcome remains largely unclear. One possible explanation for this difference is a possible age difference between female and male patients at presentation, as prior studies have shown there to be a difference in age of presentation regarding gender in different subtypes of PA, most notably in prolactinoma, as mentioned above [63, 94]. In the studies included in this analysis, the two biggest studies Tiwari et al. [83] and Yasuda et al. [93] found lower age to be a risk factor for developing postoperative DI but neither of those studies analyzed or reported age of presentation by gender and its possible influence on this higher risk of postoperative DI at a lower age [83, 93]. So while there may be gender differences in age of presentation and both lower age at presentation and female gender have now been linked to higher rates of DI, it remains unclear if the two are linked or independent risk factors for postoperative DI. Additionally, the underlying reasons for a higher incidence of DI in both remain unclear while both a more aggressive surgical approach in younger patients and a smaller pituitary gland with a therefore higher vulnerability to resection in females have been discussed [83, 93]. While in our analysis we found a low heterogeneity of included studies, it must be stated that definitions of DI did differ significantly between included studies, most noticeably in the two biggest included studies Tiwari et al. [83] and Yasuda et al. [93] While in Yasuda et al. [93] DI was defined as “(1) polyuria: urinary flow greater than 250 ml/h for more than 2 h and (2) urinary hypoosmolarity: defined as a urinary density less than 1005,” Tiwari et al. [83] simply defined DI as a prescription for desmopressin at the time of discharge. While considering that patients were operatively treated and hospitalized for PA adenoma and it is therefore highly likely that a prescription of desmopressin in this circumstance will have meant postoperative DI, we cannot be sure that in Tiwari et al. [83] the diagnostic criteria of DI was homogeneous in their institution over included years, that this DI is a new postoperative phenomenon, or that, in fact, desmopressin was prescriped for DI in the first place [22, 30, 54, 60, 71, 72].

The levels of preoperative prolactin did not differ significantly between male and female patients, although this part of the meta-analysis has to be carefully interpreted due to the high heterogeneity, low sample size, and due to the sensitivity of meta-analyses of mean differences toward non-normal distributions of the source data, which could not be judged from the original publications. Within the literature on medically treated prolactinomas, the studies of Delgrange et al. [24], Khare et al. [46], and Nishioka et al. [66] all reported a significantly higher level of pre-treatment prolactin in male than female patients and with that a strongly correlating tumor size. Reasons for these bigger tumors in male patients are controversial and might be explained by either a longer delay of diagnosis due to fewer early symptoms of hyperprolactinemia or the greater proliferation potential of these tumors in male patients [24, 46, 66]. As explained above, the meta-analysis on prolactin levels that was possible from the included studies has to be carefully considered. In addition, while in most cases medical treatment is the appropriate initial treatment for prolactinoma [5, 66, 69] and in the abovementioned studies all patients were at least initially treated medically, our analysis only included patients that underwent transsphenoidal PA surgery, and therefore, levels of serum prolactin might be different than in patients that are initially or purely treated medically.

In nearly all the analyzed endpoints, except for the rate of DI, there was high heterogeneity of included studies. This is most likely due to the very different results of included studies regarding the impact of gender on analyzed outcomes, which in turn might be due to the small to very small sample sizes of most included studies. While high heterogeneity is not particularly desired, it could not be avoided in our analysis due to the very little available literature on the influence of gender on PA surgery.

Most included studies (32/40) were given a GRADE rating of “very low” with the remaining studies rated “low” (Table 1). This rating again was due to the low to very low number of either patients overall or outcomes reported by gender in included studies.

In terms of certainty assessment, the risk of bias was rated as not serious in all analyzed outcomes according to the GRADE framework [37]. Inconsistency was rated serious in analyzed outcomes of biochemical remission and GTR. In both outcomes, this was due to a high heterogeneity and low sample size of included studies as mentioned above. In postoperative DI, inconsistency was rated as not serious, as the analysis showed a low heterogeneity. In preoperative levels of prolactin, inconsistency showed to be very serious, due to the abovementioned high heterogeneity, low sample size, and sensitivity of meta-analyses of mean difference toward non-normal distributions of data. Indirectness was rated as not serious in all analyzed outcomes according to the GRADE framework [35]. Imprecision was rated as serious in both postoperative biochemical remission and DI, as very serious in postoperative GTR, and as extremely serious in preoperative levels of prolactin according to the GRADE framework [36], while in all outcomes, plausible residual confounding like age, comorbidities, and surgical indication would reduce the demonstrated effect. Overall, according to the GRADE framework, the analysis of postoperative DI had a low overall certainty, while the analysis of postoperative biochemical remission, GTR, and preoperative levels of prolactin all had a very low overall certainty (Table 2). This overall level of certainty is not surprising considering there were relatively few studies reporting outcomes by gender, a low to very low sample size of those who did, and a therefore relatively low sample size in all analyzed outcomes with most, except for postoperative DI, showing high heterogeneity.

Although our meta-analysis did not find a statistically significant difference of gender in postoperative biochemical remission, rate of GTR, or preoperative prolactin levels, these findings do not establish that there is no difference of gender in these outcomes at all. As this study searched for differences in all transsphenoidal pituitary adenoma surgery regardless of specific subtype, this generalization can lead to misleading findings and possible influences of gender in these subtypes may be overlooked. Furthermore, we cannot assure the homogeneity of included studies concerning age, comorbidities, and surgical indication. Nonetheless, our analysis found a higher rate of postoperative DI in female than male patients and while a possible influence of gender on postoperative biochemical remission, rate of GTR, or preoperative prolactin could not be found in our meta-analysis, still, in single studies, it appears that gender may have an influence on outcomes after pituitary surgery.

While there was a significantly higher rate of postoperative DI in female patients after endoscopic or microscopic transsphenoidal PA surgery, our analysis of the influence of gender on postoperative biochemical remission, rate of GTR, and preoperative prolactin levels did not demonstrate a statistically significant effect. Further research and studies with larger sample sizes and considering PA subtypes and different age groups (premenopausal vs. postmenopausal) are needed to establish a clear understanding of their impact on PA surgery. While reporting data stratified by gender and sexual hormones would be relatively easy, there is still little data reported as such and its significance remains to be examined. Future studies concerning PA surgery should report their data by gender or sexual hormones and ideally further assess their impact on PA surgery.

## Limitations

The main limitation of this study is the general lack of gender-specific data reported in publications and the therefore relatively small sample size, although reporting data by gender would be simple and might lead to new evidence regarding gender sciences and its impact on neurosurgery and medicine as a whole.

Furthermore, we analyzed all PA treated with endoscopic or microscopic transsphenoidal surgery as a group and, because of the limited reporting and small sample sizes, did not specify PA by subtype. Gender and sexual hormones might have varying influences on surgical outcomes of different subgroups and a generalization of all PA may lead to misleading results. Additionally, the homogeneity of included studies concerning age, comorbidities, and surgical indication cannot be assured.

Another limitation of this analysis is that most included studies classified PA according to clinical phenotype. While as mentioned our analysis did not differ between subtypes of PA, there might have been differences in the classification of PA in included studies as PA subtypes defined as its clinical phenotype is not always identical to respective pathological studies, and the classification of tumors may have changed over the time period in which included studies were published.

Moreover, we cannot be sure that included studies homogenously defined reported outcomes as, for example, and as mentioned before, postoperative DI was not homogenously defined in all included studies.

Additionally, due to the small sample sizes, there is high heterogeneity in our analyses, and as a meta-analysis, there might be inherent publication bias in this study.

## Conclusions

After an extensive literature search, we analyzed 40 studies regarding the influence of gender on endoscopic or microscopic transsphenoidal PA surgery. In our meta-analysis, there was a significantly higher rate of postoperative DI in female patients after endoscopic or microscopic transsphenoidal PA surgery, and although there was some data in isolated studies suggesting the influence of gender on postoperative biochemical remission, rate of GTR, and preoperative prolactin levels, these findings could not be confirmed in this meta-analysis and demonstrated no statistically significant effect. Further research is needed and future studies concerning PA surgery should report their data by gender or sexual hormones and ideally further assess their impact on PA surgery.

## Supplementary Information

Below is the link to the electronic supplementary material.

Supplementary file1 (PDF 184 KB)

Supplementary file2 (PDF 115 KB)

## Data Availability

All data supporting the findings of this study are available within the paper and its Supplementary Information.

## Abbreviations

PA: Pituitary adenoma
NFPA: Non-functioning pituitary adenoma
GTR: Gross total resection
STR: Subtotal resection
PRISMA: Preferred Reporting Items for Systematic Reviews and Meta-Analyses
GRADE: Grading of Recommendations, Assessment, Development, and Evaluations
OR: Odds ratio
DI: Diabetes insipidus
ACTH: Adrenocorticotropic hormone
TSH: Thyroid-stimulating hormone
FSH: Follicle-stimulating hormone
LH: Luteinizing hormone
GH: Growth hormone
SIADH: Syndrome of inappropriate antidiuretic hormone secretion
