# Supplementary material for: Influence of gender and sexual hormones on outcomes after pituitary surgery: a systematic review and meta-analysis
Source: Acta Neurochir (Wien). 2023 Aug 9;165(9):2445–60. doi: 10.1007/s00701-023-05726-z (PMC10477253; doi:10.1007/s00701-023-05726-z)
Supplement: Supplementary file 1 — Supplementary file1 (PDF 184 KB) [file 701_2023_5726_MOESM1_ESM.pdf]

Influence of gender and sexual hormones on outcomes after pituitary surgery:

A systematic review and meta-analysis

Journal: Acta Neurochirurgica

Sven Theiler[1], BMed; Saskia Hegetschweiler[1], BMed; Victor E. Staartjes[1], MD, PhD; Antonio Spinello[1], MD; Giovanna Brandi[2], MD;

Luca Regli[1], MD; Carlo Serra[1], MD

1: Machine Intelligence in Clinical Neuroscience (MICN) Laboratory, Department of Neurosurgery, Clinical Neuroscience Center, University Hospital Zurich, University of Zurich, Zurich, Switzerland

2: Institute for Intensive Care, University Hospital Zurich, University of Zurich, Zurich, Switzerland

Corresponding Author

Carlo Serra, MD

Senior Physician and Associate Professor

Department of Neurosurgery

University Hospital Zürich

Frauenklinikstrasse 10, 8091 Zürich

Tel +41 44 255 2660

Fax +41 44 255 4505

E-Mail carlo.serra@usz.ch

Supplementary Table 1: Details of not analyzed endpoints

Year	First Author	No. Total	No. male	No. female	Male PreTesto	Female PreTesto	Male PreEstro	Female PreEstro	Male HyperPRL	Female HyperPRL	Male FSH/LH def	Female FSH/LH def	Male GH def	Female GH def	Male ACTH def	Female ACTH def	Male TSH def	Female TSH def	Male panhypo	Female panhypo	Male any endo def	Female any endo def	Male SIADH	Female SIADH
2018	Antunes	69	36	33																				
2020	Asha	81	36	45																				
2019	Aydin	27	16	11																				
2010	Baumann	6	5	1											4	0	3	0						
2010	Bellut	37	24	13																				
2020	Bora	85	26	59																				
2010	Campbell	26	14	12																				
2016	Cote	7	5	2																				
2019	Fallah	80	61	19																				
2009	Fomekong	40	3	37																				
2010	Hofstetter	24	13	11																				
2011	Jane	60	33	27																				
2017	Kim	134	60	74																				
2020	Kim	31	13	18																				
2012	Ku	282	121	161																				
2016	Ku	187	83	104																				
2019	Little	169	99	70																	9	5		
1997	Mason	10	5	5																				
2017	Mayberg	81	14	67																				
2017	Park	463	203	260																				
2000	Sanno	16	4	12																				
2019	Serban	61	12	49																				
2017	Shin	50	11	39																				
2018	Taghvaei	68	36	32																				

2022	Tiwari	210	104	106															60	65				
2019	Wang	87	34	53																				
2013	Wilson	14	6	8																				
2015	Yamada	252	37	215																				
2018	Yoo	79	22	57					9	5														
2015	Zieliński	10	3	7																				

No. total = total number of patients, No. male = total number of male patients, No. female = total number of female patients, Male PreTesto = Preoperative levels of testosterone in male patients, Female PreTesto = preoperative levels of testosterone in female patients, Male PreEstro = Preoperative levels of estrogen in male patients, Female PreEstro = Preoperative levels of estrogen in female patients, Male HyperPRL = preoperative levels of hyperprolactinaemia in male patients, Female HyperPRL = preoperative levels of hyperprolactinaemia in female patients, Male FSH/LH def = Number of male patients with postoperative FSH and/or LH deficit, Female FSH/LH def = Number of female patients with postoperative FSH and/or LH deficit, Male GH def = Number of male patients with postoperative GH deficit, Female GH def = Number of female patients with postoperative GH deficit, Male ACTH def = Number of male patients with postoperative ACTH deficit, Female ACTH def = Number of female patients with postoperative ACTH deficit, Male TSH def = Number of male patients with postoperative TSH deficit, Female TSH def = Number of female patients with postoperative TSH deficit, Male panhypo = Number of male patients with postoperative panhypopituitarism, Female panhypo = Number of female patients with postoperative panhypopituitarism, Male any endo def = Number of male patients with any postoperative endocrinological deficit, Female any endo def = Number of female patients with any postoperative endocrinological deficit, Male SIADH = Number of male patients with postoperative SIADH, Female SIADH = Number of female patients with postoperative SIADH.
