# Supplementary material for: Influence of gender and sexual hormones on outcomes after pituitary surgery: a systematic review and meta-analysis
Source: Acta Neurochir (Wien). 2023 Aug 9;165(9):2445–60. doi: 10.1007/s00701-023-05726-z (PMC10477253; doi:10.1007/s00701-023-05726-z)
Supplement: Supplementary file 2 — Supplementary file2 (PDF 115 KB) [file 701_2023_5726_MOESM2_ESM.pdf]

Influence of gender and sexual hormones on outcomes after pituitary surgery: A systematic review and meta-analysis

Journal: Acta Neurochirurgica

Sven Theiler[1], BMed; Saskia Hegetschweiler[1], BMed; Victor E. Staartjes[1], MD, PhD; Antonio Spinello[1], MD; Giovanna Brandi[2], MD; Luca Regli[1], MD; Carlo Serra[1], MD

1: Machine Intelligence in Clinical Neuroscience (MICN) Laboratory, Department of Neurosurgery, Clinical Neuroscience Center, University Hospital Zurich, University of Zurich, Zurich, Switzerland

2: Institute for Intensive Care, University Hospital Zurich, University of Zurich, Zurich, Switzerland

Corresponding Author

Carlo Serra, MD

Senior Physician and Associate Professor

Department of Neurosurgery

University Hospital Zürich

Frauenklinikstrasse 10, 8091 Zürich

Tel +41 44 255 2660

Fax +41 44 255 4505

E-Mail carlo.serra@usz.ch

Supplementary Table 2: Exact search strategies stratified by database

Database	Search terms	Exceptions	Strategy	Results
Pubmed/MEDLINE	Pituitary; adenoma; surgery; resection; transsphenoidal; gender; sex; male; female; prolactin; testosterone; estrogen; gross total resection; GTR; deficit; endocrine; endocrinological; biochemical	From january 1st, 1990 onwards	pituitary AND adenoma AND (surgery OR resection OR transsphenoidal) AND (gender OR sex OR male OR female OR prolactin OR testosterone OR estrogen OR oestrogen) AND (gross total resection OR gtr OR deficit OR endocrine OR endocrinological OR biochemical)	2772

No duplicates
